# Purified Lesser weever fish venom (*Trachinus vipera*) induces eryptosis, apoptosis and cell cycle arrest

**DOI:** 10.1038/srep39288

**Published:** 2016-12-20

**Authors:** Myriam Fezai, Chaker Slaymi, Mossadok Ben-Attia, Florian Lang, Mohamed Jemaà

**Affiliations:** 1 Laboratory of Biomonitoring of the Environment (LR01/ES14), Faculty of Sciences of Bizerte, Tunis street, 7021 Zarzouna, Bizerte, Tunisia; 2 University of Carthage, Amilcar avenue 77, 1054 Tunisia; 3 Department of Cardiology, Vascular Medicine and Physiology, University of Tuebingen, Gmelinstr. 5/Otfried-Mueller-Str. 10, D-72076 Tuebingen, Germany; 4 Centre de Recherche de Biochimie Macromoléculaire - CNRS, UMR 5237, Mende 1919, 34293 Montpellier, France; 5 University of Montpellier, Auguste Broussonet street 163, 34090 Montpellier, France

## Abstract

Accidents caused by the sting of *Trachinus vipera* (known as Lesser weever fish) are relatively common in shallow waters of the Mediterranean. Symptoms after the sting vary from severe pain to edema or even tissue necrosis in some cases. Here we show that purified Lesser weever fish venom induces eryptosis, the suicidal erythrocyte death, and apoptosis of human colon carcinoma cells. The venom leads to erythrocyte shrinkage, phosphatidylserine translocation and increased intracellular Ca^2+^, events typical for eryptosis. According to mitochondrial staining cancer cells dyed after the activation of the intrinsic apoptotic pathway. *Trachinus vipera* venom further causes cell cycle arrest.

Tunisia’s coast is about 1500 km in the heart of the Mediterranean Sea. Envenomations caused by the sting of *Trachinus vipera* fish (best known as Lesser weever or “Bellem-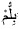
” in Tunisian dialect) are relatively common in shallow waters of the Mediterranean and North sea, especially during the spring and summer^1^,^2^. The Lesser weever fish is the smallest specimen from the Trachinidae piscine family^2^,^3^,^4^. The dorsal spines of the fish, made of clusters of glandular cells, harbour the venom^5^,^6^. Accidents following the sting of the weever fish, generally in the lower extremities of fishermen and tourists, cause common symptoms especially small bite with evident erythema. Erythema spreads and oedema appears within few hours with numbness feeling. Inflammation can last for two weeks or more, and the affected limb can be highly limited in mobility^2^,^4^,^5^,^7^,^8^,^9^. Other systemic symptoms can occur i.e. nausea and headache^10^, tissue necrosis^4^,^11^, Raynaud’s Phenomenon^12^ and fatality have been recorded^5^. The previous studies on the Lesser weever fish were mainly addressing the ecology, biology, anatomy of the venomous apparatus of the fish and some clinical case reports following the sting of the animal. However, so far, there are no published findings outlining the pharmacological potential of the Lesser weever fish venom, in contrast to the Greater weever fish venom (*Trachinus Draco*) of the same family, shown in few studies to exert cardiovascular hypotensive, cytolytic, and neuromuscular effects^1^,^13^,^14^,^15^. It is timely, therefore, to explore the properties of the Lesser weever venom, especially cell death, based on the necrotic effect of the sting described in several reports.

In this study, we aimed to investigate the potential pharmacological effect of the *Trachinus vipera* venom on two models of cells/tissue namely: Human Erythrocytes (red blood cells) and Human Colon Carcinoma HCT116 cells.

## Results

### The *Trachinus vipera* venom stimulates suicidal erythrocyte death

**Figure 1:**
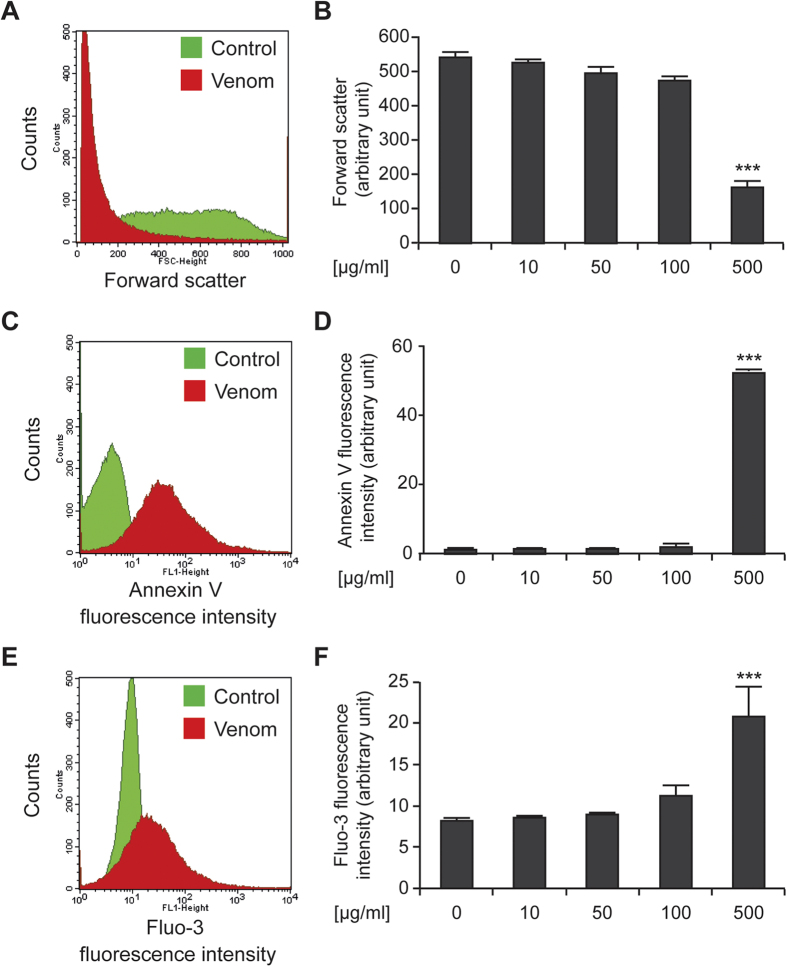
*Trachinus vipera* venom induces eryptosis. (**A**,**B**) Effect of venom on the erythrocytes size. Erythrocytes were maintained in Ringer solution followed by treatment or not for 48 h with 10 to 500 μg/ml of venom. The forward scatter of erythrocytes was estimated by flow cytometry. (**A**) Illustrates representative dot plots (control was labeled in green and 500 μg/ml venom in red), while (**B**) report quantitative data. Data are reported as means ± SEM (n = 9). (**C**,**D**) Effect on phosphatidylserine exposure. Erythrocytes (control and treated ones) was labeled with annexin-V for the assessment of apoptosis-associated parameters (phosphatidylserine exposure). (**B**) Illustrates representative dot plots (control was labeled in green and 500 μg/ml venom in red), while (**C**) report quantitative data. Data are reported as means ± SEM (n = 9). (**E**,**F**) Effect of venom on erythrocyte Ca^2+^ activity. Erythrocytes (control and treated ones) was labeled with Fluo3-AM for the assessment of erythrocyte cytosolic Ca^2+^ concentration. (**E**) Illustrates representative dot plots (control was labeled in green and 500 μg/ml venom in red), while (**F**) report quantitative data. Data are reported as means ± SEM (n = 9). ***(*p* < 0.001) indicate significant difference as compared with non-treated erythrocyte (ANOVA).

The envenomation by *Trachinus vipera* is caused by the inoculation of the venom following the spines´sting^6^ (Fig. S1) and thus can have direct contact with blood. We explored first the effect of the *Trachinus vipera* purified venom on erythrocytes death and in particular eryptosis (Fig. 1). This suicidal death is characterized by cell shrinkage and phosphatidylserine translocation to the cell surface^16^. To this end, erythrocytes were incubated for 48 h in Ringer solution without or with *Trachinus vipera* venom (10–500 μg/ml). In order to estimate the alterations of cell volume, forward scatter was determined in flow cytometry and as illustrated in (Fig. 1A,B), the exposure to the venom was followed by a significant decrease of forward scatter (at 500 μg/ml). Accordingly, venom administration was followed by erythrocytes shrinkage. Phosphatidylserine exposing erythrocytes were identified utilizing annexin-V-binding and as shown in (Fig. 1C,D), at 48 h, the percentage of annexin-V-binding erythrocytes increased particularly at 500 μg/ml. Thus, venom administration led to erythrocyte cell membrane scrambling with translocation of phosphatidylserine to the cell surface. Since both, cell shrinkage and cell membrane scrambling with phosphatidylserine translocation to the cell surface are stimulated by increase of cytosolic Ca^2+^ activity ([Ca^2+^]_i_), further experiments estimated the effect of *Trachinus vipera* venom on [Ca^2+^]_i_. To this end, erythrocytes were loaded with Fluo3-AM and the Fluo3 fluorescence was determined by flow cytometry. The exposure of the erythrocytes to venom was followed by an increase of Fluo3 fluorescence at 500 μg/ml (Fig. 1E,F). Consequently, the venom increased the concentration of cytosolic Ca^2+^. These findings disclose that Lesser weever fish venom triggers eryptosis.

### The *Trachinus vipera* venom kills HCT116 cells *in vitro*

**Figure 2:**
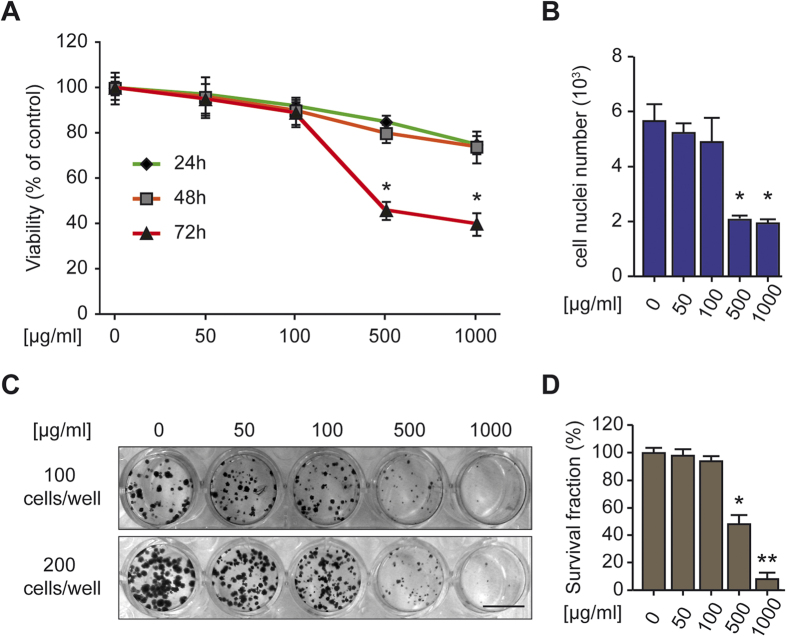
The antiproliferative effect of *Trachinus vipera* venom on HCT116 cells. (**A**) Dose-response curves for the effect of *Trachinus vipera* venom on the viability of HCT116. Cells were treated with indicated concentrations of the venom for 3 days and cell viability was determined using a WST-1 assay. Green curve represents the effect on 24 h, orange on 48 h and red on 72 h respectively, (mean ± SEM; n = 3). *p < 0.05, as compared with non-treated cells. (**B**) Cell nucleus count. HCT116 cells were treated with indicated doses of venom for 72 h and then DAPI stained. The number of nuclei was counted for each condition. Quantitative results are reported. (**C**,**D**) Long effect potential. HCT116 cells were seeded at low cell density. After 24 h cells were treated for 1 week with the indicated concentration of venom. Colonies were fixed and stained with cristal violet for count. Representative plate (**C**) as well as quantitative data upon normalization to plating efficiency (**D**) (mean ± SEM; n = 3) are shown. *p < 0.05 and **p < 0.01 (Student’s t-test), as compared with non-treated cells. Scale bar = 1 cm.

To investigate the effect of *Trachinus vipera* purified venom on cells *in vitro*, we first tested different concentrations on HCT116 cells using the WST-1 viability assay. Cells were seeded in the 96 well plates and then treated with the Lesser weever fish venom (50, 100, 500 and 1000 μg/ml) for 24, 48 and 72 h. HCT116 cells displayed a growth inhibition in response to the treatment at 72 h and with an IC_50_ of 460 +/− 40 μg/ml of *Trachinus vipera* venom (Fig. 2A). In a separate series, a cells count was performed. Cells were seeded on coverslips glasses and treated. After 72 h cells were fixed and nuclei were stained with Hoechst. The nuclei count using image J showed a similar result to WST-1 test, i.e. 500 μg/ml treated wells/cells contained (after wash and fixation) about half of the cells number counted in control condition (Fig. 2B). Consistent with these results, 500 μg/ml (and more with 1000 μg/ml) reduced the clonogenic potential of HCT116 cells, as determined in clonogenic assays, a test reflecting long term effect/toxicity^17^ (Fig. 2C,D). These results confirm that the venom of *Trachinus vipera* starting at 72 h/500 μg/ml exert toxic and anti-proliferative effects on cancer cells.

### Mechanisms underlying cell death induced by *Trachinus vipera* venom

**Figure 3:**
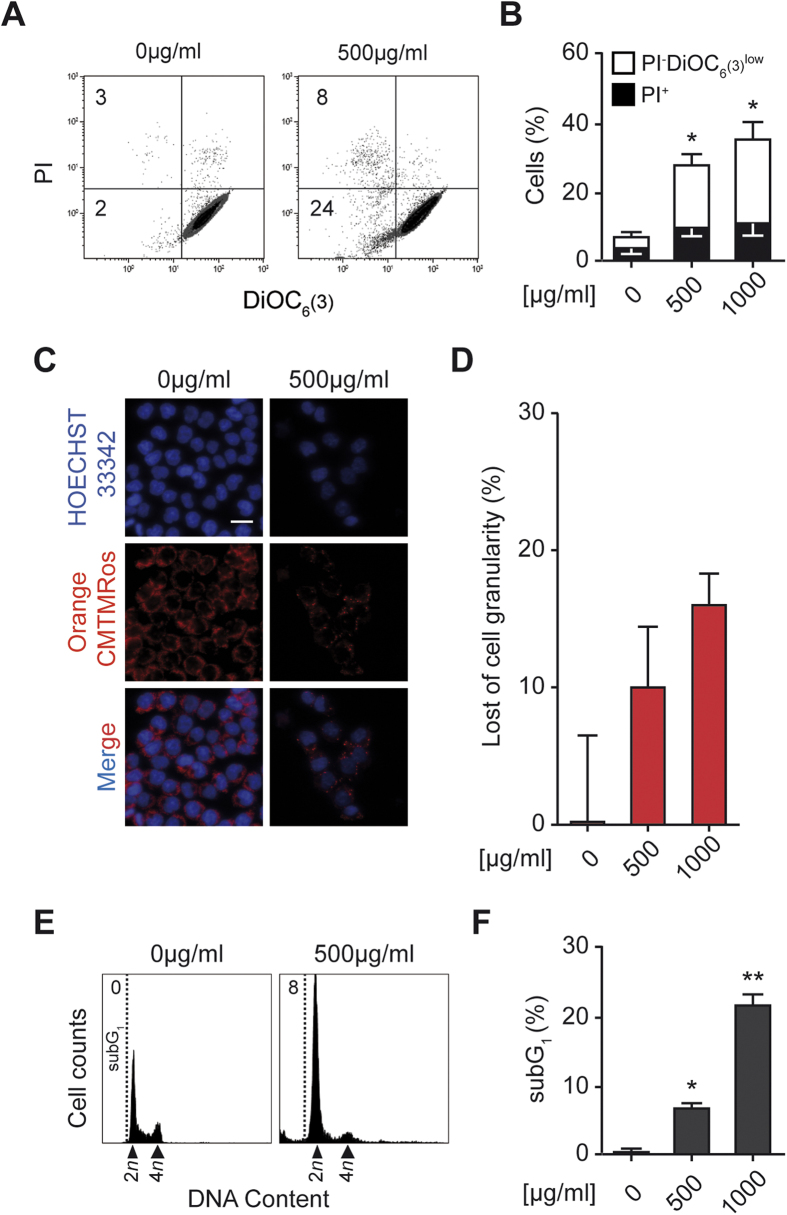
*Trachinus vipera* venom kills HCT116 cells by apoptosis. (**A**,**B**) Cell death characterization. Human colon carcinoma HCT116 cells were maintained in control conditions or treated for 72 h with 500 μg/ml of venom. Cells were then co-stained with DiOC_6_(3), and propidium iodide (PI) for the assessment of apoptosis-associated parameters. (**A**) Illustrates representative dot plots (numbers indicate the percentage of cells found in the corresponding quadrant), while (**B**) report quantitative data. White and black columns illustrate the percentage of dying (PI^−^ DiOC_6_(3)^low^) and dead (PI^+^) cells, respectively. Data are reported as means ± SEM (n = 3). (**C**,**D**) Labeling mitochondria. HCT116 cells were maintained in control conditions or treated for 72 h with 500 μg/ml of venom. After 30 minutes labeling with the MitoTracker Orange CMTMRos, cells were fixed and stained with Hoechst to visualize nucleus. Representative immunofluorescence microphotographs of untreated *vs* treated cells are shown in (**C**) (scale bar = 10 μm), while quantitative results (means ± SEM; n = 3) are reported in (**D**). (**E**,**F**) Staining of micronucleus. HCT116 cells were maintained in control conditions or treated for 72 h with 500 μg/ml of venom, followed by Hoechst 33342 staining (to quantify cells manifesting apoptotic DNA degradation, subG1). (**E**,**F**) Illustrate representative histograms and quantitative data (mean ± SEM; n = 3), respectively. *p < 0.05 and **p < 0.01 as compared with non-treated cells.

To understand how Lesser weever fish purified venom induces HCT116 cells mortality, we performed a cells co-staining with the vital dye propidium iodide (PI) and the mitochondrial transmembrane potential (Δψ_m_) sensor DiOC_6_(3). Indeed, the PI cell incorporation shows loss of cell membrane integrity and in consequence cell death while loss of mitochondrial transmembrane potential is a sign of early stage apoptosis. The combination of these two parameters is an indication of cell death^18^. The frequency of dying (DiOC_6_(3)^low^ PI ^low^) and dead (DiOC_6_(3)^low^ PI ^high^) cells analyzed by flow cytometry was markedly increased among venom treated cells to reach about 32% at 500 μg/ml of venom. The pre-apoptotic fraction (DiOC_6_(3)^low^) constitutes 75% of the total death. (Fig. 3A,B). We confirmed the FACS data with microscopy. Indeed, living labeled cells with the MitoTracker^®^ Orange CMTMRos (to show the loss of mitochondrial transmembrane potential on microscope) were co-stained with Hoechst to visualize the nucleus after fixation and the loss of granularity was evaluated^19^. Treated cells lost about 10 to 20% of granularity, which is corresponding to the proportion of cells that entered early apoptosis (Fig. 3C,D). Moreover, the analysis of the cell cycle profile, showed an appearance of a sub-diploid population (Sub G_1_) corresponding to apoptotic bodies^20^ in the treated compared to control cells (Fig. 3E,F). Altogether, the marked loss of mitochondrial transmembrane potential and the accumulation of apoptotic bodies in treated cells suggest that the *Trachinus vipera* venom induces death after activation of the mitochondrial pathway of apoptosis.

### *Trachinus vipera* venom cause cell cycle arrest

**Figure 4:**
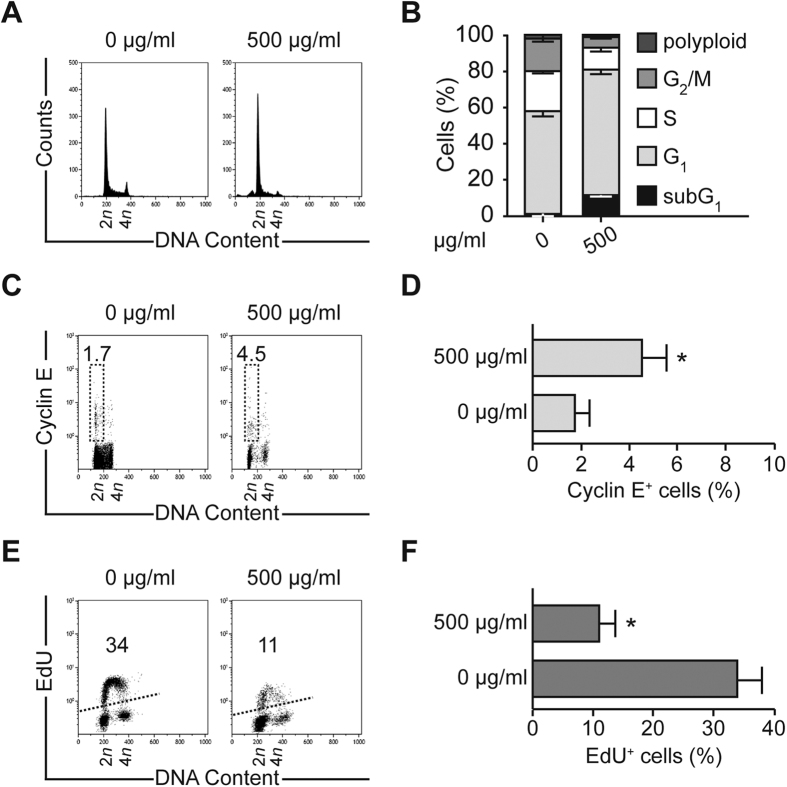
*Trachinus vipera* venom induces accumulation in G_1_ phase of cell cycle and stops DNA synthesis. (**A**,**B**) Cell cycle analysis. Human colon carcinoma HCT116 cells were exposed to 500 μg/ml *Trachinus vipera* venom for 72 h followed by the cytofluorometric assessment of cell cycle distribution. (**A**,**B**) report representative cell cycle distributions and quantitative data (mean ± SEM; n = 3). (**C**,**D**) G_1_ phase staining. HCT116 cells were cultured in the presence of venom for 72 h, fixed and then labeled with an antibody recognizing cyclin E, which accumulates essentially in the G_1_ phase. Cells were acquired using cytofluorometry. Representative scatter plots (**C**) and quantitative results (**D**) (mean ± SEM; n = 3) are shown. (**E**,**F**) DNA synthesis evaluation. HCT116 cells were cultured in the presence of 500 μg/ml *Trachinus vipera* venom and after 72 h labeled with the thymidine analog EdU, which selectively stains cells in the S phase of the cell cycle. Cell were fixed and acquired by cytofluorometry to evaluate the level of EdU incorporation. Representative scatter plots (**E**) and quantitative results (**F**) (mean ± SEM; n = 3) are shown. Numbers indicate the percentage of cells found in each quadrant. *p < 0.05 (Student’s t-test), as compared with non-treated cells.

**Figure 5:**
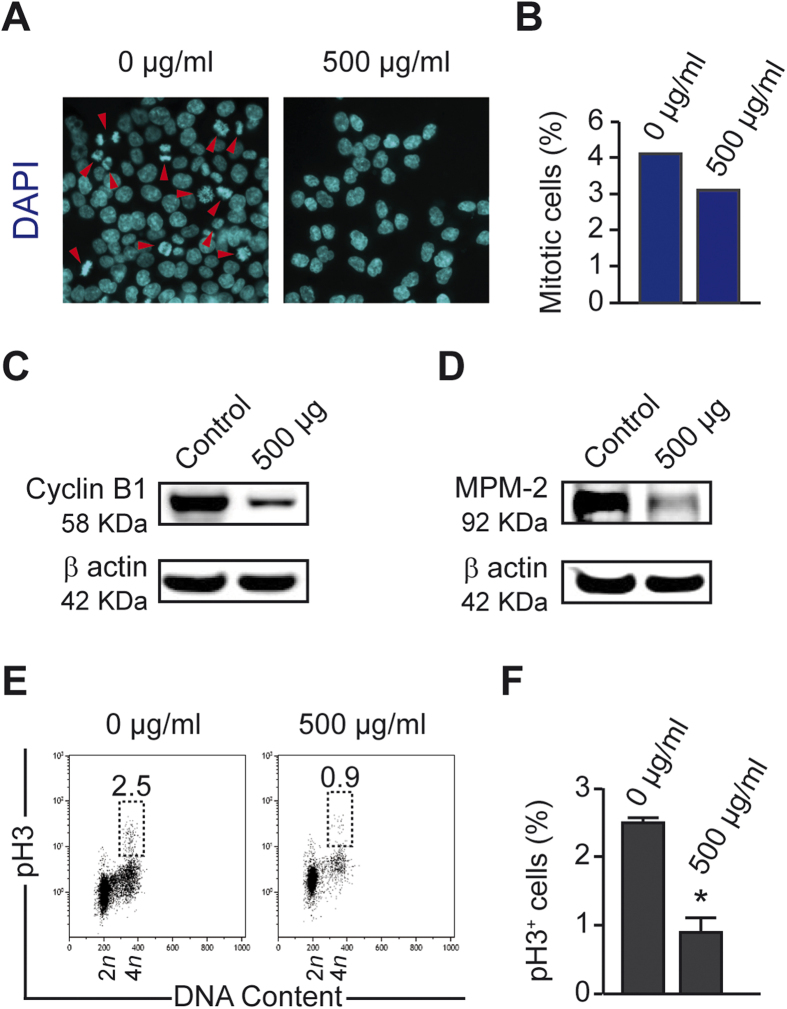
*Trachinus vipera* venom inhibits the mitotic entry of HCT116 cells. (**A**,**B**) Estimation of mitosis percentage. HCT116 cells were treated with 500 μg/ml *Trachinus vipera* venom and fixed then labeled with DAPI at 72 h. Cells were visualized and counted using a fluorescence microscope. Representative immunofluorescence microphotographs of untreated *vs* treated cells are shown in (**A**) (scale bar = 10 μm), while quantitative results (means ± SEM; n = 3) are reported in (**B**). Labeling mitotic markers. (**C**–**F**) Cells were subjected to immunoblotting with an antibody recognizing cyclin B1, which accumulates starting from the early G_2_ phase through the end of mitosis (**C**) or with an antibody recognizing the mitotic marker MPM-2, which are phosphorilated during the mitosis (**D**). Fixed cells are acquired after staining with an antibody recognizing the phospho-histone H3 (pH3), accumulated during mitosis. Representative scatter plots (**E**) and quantitative results (**F**) (mean ± SEM; n = 3) are shown. Numbers indicate the percentage of cells found in each quadrant. *p < 0.05 (Student’s t-test), as compared with non-treated cells.

We further investigated the cell cycle perturbation after treatment with the fish venom. Cells were treated with the purified venom (500 μg/ml) and analyzed compared to non-treated cells using flow cytometry. A clear cycle arrest on G_1_ phase of treated cells was noted. Indeed, the profile showed an accumulation of 20% more cells in G_1_ at 72 h of treatment with venom (Fig. 4A,B). Moreover, in response to treatment, cells exhibited a marked increase (more than twice) of cyclin E protein abundance (the cyclin E accumulated in G_1_ phase and early S phase) (Fig. 4C,D). On the other hand, the incorporation of the thymidine analog 5-ethynyl-20-deoxyuridine (EdU) into DNA was significantly reduced by the treatment, inducing decrease in cycling and duplicating (Fig. 4E,F). At the same time, the mitotic index was estimated with or without treatment. The simple count of mitotic cells with microscopy showed a decrease in the percentage of mitosis (about 25% comparing to control) (Fig. 5A,B). Also, the treated cells presented a decline in cyclin B1 (the cyclin associated with the G_2_/M phase) (Fig. 5C) as well as the mitotic-specific phosphoepitope MPM2 (a specific marker of mitotic entry) (Fig. 5D). The phosphorylation of histone H3, another indicator of ongoing mitosis, is also reduced in treated compared to control cells (Fig. 5E,F). Collectively, the data suggest cell cycle arrest and accumulation of treated HCT116 cells in an interphasic stage.

## Discussion

Driven by the incidence and pattern of injuries caused by *Trachinus vipera* venom, we decided to investigate the pharmacological potential of the Lesser weever fish venom in two models that are erythrocytes and Colon Carcinoma HCT116 cells. Fish specimens were collected and the venom was extracted from the dorsal fins by mechanical techniques and dialysis (Fig. S1). The venom induced apoptosis of human colon carcinoma HCT116 cells. According to the WST-1 test, cell count and Clonogenic assays, Lesser weever fish venom compromizes the survival of HCT116 cancer cells. The WST-1 assay detects the production of formazan activity and thus shed light on the metabolism of the cells^21^. Moreover, cell count and Clonogenic assay demonstrate the potential of the venom to arrest cell proliferation (Fig. 2). The venom clearly provokes apoptosis. There are two main pathways that lead to apoptosis: the death receptor pathway initiated by tumor necrosis factor receptors which is the extrinsic pathway^22^ and the mitochondrial or the intrinsic pathway, which involves mitochondria and Bcl-2 family members^23^. The intrinsic pathway is initiated by loss of the mitochondrial transmembrane potential which leads to opening of the mitochondrial permeability transition pores and release of effectors including cytochrome *c* and apoptosis inducing factor from the mitochondria into the cytosol. Cytochrome *c* triggers the proteolytic activity of caspase-3 and caspase-9 in the cytosol, then the activation of caspases degrade (poly [ADP-ribose] polymerase PARP) and caspase-activated-DNase, which initiates DNA degradation^24^. Our findings, in HCT116, demonstrated that the Lesser weever fish venom kills cells by the activation of the intrinsic pathway of apoptosis (loss of mitochondrial transmembrane potential and membrane integrity and the degradation of nucleus) (Fig. 3). The effect of the purified venom was also tested on erythrocytes. Red blood cells (erythrocytes) lack nuclei and mitochondria, and as consequence should be insensitive to the suicide death provoked by the mitochondrial pathway of apoptosis^25^. However, erythrocytes can undergo suicide *via* another pathway. This particular death is baptized eryptosis, characterized by cell shrinkage, blebbing and cell membrane scrambling with phosphatidylserine translocation to the cell surface. Eryptosis can be induced by ceramide^26^, oxidative stress^16^, energy depletion^16^, as well as stimulation of some kinases including casein kinase 1α, Janus-activated kinase JAK3, protein kinase C, and/or p38 kinase^16^. Another important trigger of eryptosis is activation of caspases. Indeed, despite the inability of red blood cells to synthesize proteins, they contain functional apoptotic caspases, in particular caspase 3^27^,^28^,^29^,^30^. Moreover, erythrocytes contain Fas, FasL, Fas-associated death domain FADD and the active caspase 8^27^,^28^. Eryptosis is induced by the activation of Fas-signaling complex (binding of transmembrane FasL to its receptor Fas or the association between FADD and Fas) in upstream of caspase 8 activation^31^,^32^,^33^. However, the main signaling pathway documented as inducer of eryptosis is increase of cytosolic Ca^2+^ activity ([Ca^2+^]_i_)^16^. Calcium can directly cause the phosphatidylserine membrane translocation (cell scrambling)^34^. Moreover, calcium can activate K^+^ channels with an efflux of KCl from the cell causing cell shrinkage^35^. In addition, increased cytosolic concentration of calcium is followed by activation of the cysteine endopeptidase calpain, which degrades membrane proteins and causes cell membrane blebbing^36^. Thus, size and granularity of erythrocytes were evaluated, the membrane phosphatidylserine exposure and the concentration of the cytosolic Ca^2+^ were assessed and we found that the treatment induces eryptosis, the suicidal erythrocyte death (Fig. 1).

In our study, we demonstrated also that treated human carcinoma cells HCT116 undergo cell cycle arrest. Indeed, the control of the progression of the cell cycle of cancer cells is an effective strategy for cancer therapy because deregulated cell-cycle control is a fundamental aspect of cancer for many common malignancies^37^. The findings outlined previously indicate that treated cells remain in G_1_ phase and exhibit a high level of Cyclin E, which is the Cyclin accumulated in late G_1_ phase of the cycle^38^. Also, the incorporation of thymidine analogue EdU dramatically decreased, which is a sign of the arrest of DNA duplication and thus disruption of cell proliferation^20^. Furthermore, after treatment, the amount of Cyclin B1 decreased, which is the Cyclin accumulated in late phase S and mitosis^39^. As well, the phosphorylation of the two epitopes MPM2 and pH3, that are markers of mitosis^40^, declined leading to a large reduction of the mitotic cells (Figs 4 and 5).

Presumably, piscine venoms generally possess few toxins among their compounds which are active^3^. Weever fish venom is a complex mixture of many substances including several peptides, proteins of high molecular weight such as kinin or kinin like substances, serotonin, adrenaline, noradrenaline, histamine, and several enzymes with a wide spectrum of biological activities^2^,^41^. The composition of *Trachinus Draco* venom is very similar to the venom of *Trachinus vipera*. It was shown to contain in addition, catecholamines and cholinesterase^2^,^3^. Consequently, synergistic actions between the components of the purified venom might possibly contribute to the eryptotic and the antitumor effects that we observed. Thus, further studies on the physico-chemical characterization, properties and functions of these venom components are needed.

This is the first report evaluating the semi-purified venom of *Trachinus vipera in vitro* on erythrocytes and HCT116 cells. The venom induces eryptosis characterized by cell shrinkage and phosphatidylserine translocation to the cell surface and Ca^2+^ entry. Moreover, Lesser weever fish venom provokes cells cycle arrest and apoptosis on HCT116 cells. Further studies must be conducted to identify the different components of this venom and their chemical structures.

## Materials and Methods

### Fish specimens

Specimens of Lesser weever (*Trachinus vipera*) were provided by the same fisherman and wholesaler from Kelibia port in the Cap Bon coast which is located North-East of Tunisia (36°51′N-11°05′E). Fishes were collected, identified, and shock-frozen and kept in −20 °C for venom extraction^14^. We do confirm that all experiments were performed in accordance with relevant guidelines and regulations.

### Venom extraction and dialysis

The venom was extracted from the dorsal fins of fish. To that end, fins were crushed 10 minutes in a mixer, and then proceeded to sonication at a frequency of 47 kHz during 30 min at 20 °C. Sonication helped to burst the lipid bilayer and to release the cells contents. Afterwards, the crushed mixture was centrifuged at 5000 rpm/10 min/4 °C. The supernatant was collected for dialysis against MilliQ water using dialysis cellulose membrane of 8 kDa cutoff. The dialysate was filtered through 0,22 μm, then lyophilized and stored at −20 °C until further use.

### Erythrocytes, cell lines and culture conditions

Fresh Li-Heparin-anticoagulated blood samples were kindly provided by the blood bank of the Universitätsklinikum Tübingen. The study is approved by the ethics committee of the University of Tübingen (184/2003 V), and we do confirm that all experiments were performed in accordance with relevant guidelines and regulations. The blood was centrifuged at 120 g for 20 min at 21 °C and the platelets and leukocytes-containing supernatant were disposed. Erythrocytes were washed in Ringer solution containing (in mM) 125 NaCl, 5 KCl, 1 MgSO4, 32 N-2-hydroxyethylpiperazine-N-2-ethanesulfonic acid (HEPES), 5 glucose, 1 CaCl2; pH 7.4. For the experiments erythrocytes were incubated *in vitro* at a haematocrit of 0.4% at 37 °C for 48 h.

Human colon carcinoma HCT116 cells, kindly provided by Bert Vogelstein, Johns Hopkins Kimmel Cancer Center, were routinely maintained in McCoy’s 5 A medium supplemented with 10% fetal calf serum (FCS), 10 mM HEPES buffer, 100 units/mL penicillin G sodium and 100 μg/mL streptomycin sulfate. Cells were seeded into the appropriate supports (6-, 12-, 24- or 96-well plates) 24 h before the beginning of experiments.

### Cytofluorometric studies

For the quantification of eryptosis, erythrocytes were incubated under the respective experimental condition, 50 μL of the cell suspension was washed in Ringer solution containing 5 mM CaCl2. Cells were stained with Annexin-V-FITC (ImmunoTools, Friesoythe, Germany) in this solution at 37 °C for 20 min under protection from light; then, forward scatter (FSC) of the cells was determined and annexin-V fluorescence intensity was measured. To measure cytosolic Ca^2+^, erythrocytes were washed in Ringer solution and then loaded with Fluo-3/AM (Biotium, Hayward, CA, USA) in the Ringer solution containing 5 μM Fluo-3/AM. The cells were incubated at 37 °C for 30 min and washed twice in the Ringer solution. The Fluo-3/AM-loaded erythrocytes were resuspended in 200 μL Ringer. Then, Ca^2+^-dependent fluorescence intensity was measured.

For the simultaneous quantification of plasma membrane integrity and mitochondrial transmembrane potential (Δψm), cells were collected and stained with 1 μg/mL propidium iodide (PI), which only incorporates into dead cells, and 40 nM 3,3′-dihexyloxacarbocyanine iodide (DiOC_6_(3), a Δψm-sensitive dye (Molecular Probes-Invitrogen), for 30 min at 37 °C. For the assessment of cell cycle distribution, cells were collected, washed once with 0.1% (w/v) D-glucose (Sigma-Aldrich) in PBS and then fixed by gentle vortexing in ice-cold 75% (v/v) ethanol (Carlo Erba Reagents) for 30 sec. After overnight incubation at −20 °C, samples were centrifuged and PBS washed to remove ethanol and stained with 50 μg/mL PI in 0.1% (w/v) D-glucose in PBS supplemented with 1 μg/mL (w/v) RNase A (Sigma-Aldrich) for 30 min at 37 °C. Afterwards, samples were incubated for at least 2 h at 4 °C before cytofluorometric analysis. For the simultaneous measurement of DNA content and histone H3 phosphorylation or cyclin E_1_ levels, cells were fixed with 75% (v/v) ethanol in PBS, permeabilized with 0.25% (v/v) Tween 20 in PBS and co-stained with 10 μM 4′,6-diamidino-2-phenylindole DAPI (Molecular Probes-Invitrogen) and a rabbit antiserum specific for phosphorylated histone H3 (rabbit polyclonal IgG1 #06–570, Millipore-Chemicon International), or a mouse antiserum specific for Cyclin E (mouse monoclonal IgG1 #551159, BD Biosciences). For the EdU assay, cells were incubated with 10 μM EdU for 30 min at 37 °C, fixed, permeabilized and stained with the Fluorescent dye azide (Click-iT^TM^ reaction cocktail, from Invitrogen) and DAPI according to the manufacturer’s instructions. Cytofluorometric acquisitions were performed by means of a FACSCalibur (BD Biosciences) or a FACScan (BD Biosciences) cytofluorometer equipped with a 70 μm nozzle or with a Gallios cytofluorometer (Beckman Coulter).

### WST-1 cell viability assay

The effect of the *Trachinus vipera* purified venom was determined by the use of WST-1 assay. HCT116 cells were seeded in 96-well plates (5000 cells/well). 24 hours later, cells were treated with different concentrations of the venom (50, 100, 500 and 1000 μg/ml) diluted in phenol red-free media (100 μl). After 24 h, 48 h and 72 h WST-1 assay reagent (Roche Applied Science, Mannheim, Germany) was subsequently added (10 μl) to each well and cells were incubated for 4 hours at 37 °C before the absorbance lecture. Each well was measured at the wavelength of 450 nm and reference wavelength of 690 nm, using a scanning multiwell spectrophotometer (Synergy 2). Statistics were calculated using Student’s t-test assuming unequal variances and the mean ± SEM is presented. Each experiment was performed in triplicate and repeated three times.

### Immunofluorescence

Immunofluorescence microscopy was performed according to conventional procedures. Briefly, cells were fixed with methanol 100% for 10 minutes at −20 °C and then washed with PBS before nucleus staining with a dilution of Hoechst 33342 (1/5000 in PBS) or 4′,6′-diamidino-2-phenylindole (DAPI, 1/5000 in PBS) 10 minutes at ambient temperature. For staining mitochondria, cells were labeled during 30 minutes with the MitoTracker Orange CMTMRos (Invitrogen) (1/1000) then fixed and stained with Hoechst to visualize the nucleus. Microphotographs with the objective 20 and 40 were taken for analyses and count. Images were analyzed with the open source software Image J (freely available from the National Institute of Health, Bethesda, MD, USA at the address http://rsb.info.nih.gov/ij/).

### Clonogenic survival assays

To evaluate clonogenic survival, HCT116 cells were seeded at two different concentrations (100 and 200 cells/well) in 24-well plates. Twenty-four hours later, cells were treated with different concentrations of the *Trachinus vipera* purified venom (50, 100, 500 and 1000 μg/ml) for one week. Colonies were then fixed/stained with an aqueous solution containing 0.25% (w/v) crystal violet, 70% (v/v) methanol and 3% (v/v) formaldehyde (Carlo Erba Reagents) and counted. Only colonies made of >30 cells were included in the quantification. For each treatment, the survival fraction (SF) was estimated according to the formula: SF = number of colonies formed/number of cells seeded.

### Immunoblotting

For immunoblotting, 25 μg of proteins were separated on 4–12% Bis-Tris acrylamide (Invitrogen) and electrotransferred to Immobilon™ membranes (Millipore Corporation, Billerica, USA). Unspecific binding sites were saturated by incubating membranes for 1 h in 0.05% Tween 20 (v:v in TBS) supplemented with 5% non-fat powdered milk (w:v in TBS), followed by an overnight incubation with primary antibodies specific for Cyclin B_1_ (mouse monoclonal IgG1 #610219, BD Biosciences), MPM-2 Ser/Thr phosphorylated (mouse monoclonal IgG1 #05-368, Millipore-Chemicon International, Temecula, CA, USA) or β actin (rabbit monoclonal IgG #4970, Cell Signaling Technology Inc.). Development was performed with appropriate horseradish peroxidase (HRP)-labeled secondary antibodies (Southern Biotech, Birmingham, USA) plus the SuperSignal West Pico chemoluminescent substrate (Thermo Scientific-Pierce).

### Statistical Analysis

Unless otherwise specified, all experiments were performed and independently repeated at least three times. All data are expressed as means ± SEM and were tested for significance using ANOVA or t-test, as appropriate. Results with *p *< 0.05 were considered statistically significant.

## Additional Information

**How to cite this article**: Fezai, M. *et al*. Purified Lesser weever fish venom (*Trachinus vipera*) induces eryptosis, apoptosis and cell cycle arrest. *Sci. Rep.* **6**, 39288; doi: 10.1038/srep39288 (2016).

**Publisher's note:** Springer Nature remains neutral with regard to jurisdictional claims in published maps and institutional affiliations.

## Supplementary Material

Supplementary Figure S1

## References

[b1] Church J. E. & Hodgson W. C. The pharmacological activity of fish venoms. Toxicon 40, 1083–1093 (2002). 12165309 10.1016/s0041-0101(02)00126-5

[b2] Bonnet M. S. The toxicology of Trachinus vipera: the lesser weeverfish. Br Homeopath J 89, 84–88 (2000). 10826448 10.1054/homp.1999.0359

[b3] Vasconcelos R., Prista N., Cabral H. & Costa M. J. Feeding ecology of the lesser weever, Echiichthys vipera (Cuvier, 1829), on the western coast of Portugal. Journal of Applied Ichthyology 20, 211–216 (2004).

[b4] Duran O. & Duran F. Y. Weever fish sting : An unusual problem. JAEM (2014).

[b5] Davies R. S. & Evans R. J. Weever fish stings: a report of two cases presenting to an accident and emergency department. J Accid Emerg Med 13, 139–141 (1996). 8653243 10.1136/emj.13.2.139 PMC1342661

[b6] Perrière C. & Goudey-Perrière F. Particularités des venins de poissons. Annales de l’institut Pasteur/Actualités 10, 253–272 (1999).

[b7] Mulcahy D. M. . Case report: Weever fish sting–an unusual cause of foot pain. Ir J Med Sci 165, 153–154 (1996). 8824014 10.1007/BF02940238

[b8] Lopacinski B., Bak M., Fiszer M., Czerniak P. & Krakowiak A. [Poisoning with weever fish venom: a case report]. Przegl Lek 66, 464–465 (2009). 20043595

[b9] Cain D. Weever fish sting: an unusual problem. Br Med J (Clin Res Ed) 287, 406–407 (1983). 10.1136/bmj.287.6389.406 PMC1548902 6135490

[b10] Erylmaz M. . Envenomation Caused by Weever Fish. Turk J Emerg Med (2006).

[b11] Dehaan A., Ben-Meir P. & Sagi A. A “scorpion fish” (Trachinus vipera) sting: fishermen’s hazard. Br J Ind Med 48, 718–720 (1991). 1931733 10.1136/oem.48.10.718 PMC1012067

[b12] Carducci M., Mussi A., Leone G. & Catricala C. Raynaud’s phenomenon secondary to weever fish stings. Arch Dermatol 132, 838–839 (1996). 8678585

[b13] Russell F. E. & Emery J. A. Venom of the weevers Trachinus draco and Trachinus vipera. Ann N Y Acad Sci 90, 805–819 (1960). 13744837 10.1111/j.1749-6632.1960.tb26424.x

[b14] Chhatwal I. & Dreyer F. Biological properties of a crude venom extract from the greater weever fish Trachinus draco. Toxicon 30, 77–85 (1992). 1375787 10.1016/0041-0101(92)90503-w

[b15] Chhatwal I. & Dreyer F. Isolation and characterization of dracotoxin from the venom of the greater weever fish Trachinus draco. Toxicon 30, 87–93 (1992). 1595081 10.1016/0041-0101(92)90504-x

[b16] Lang K. S. . Mechanisms of suicidal erythrocyte death. Cell Physiol Biochem 15, 195–202 (2005). 15956782 10.1159/000086406

[b17] Munshi A., Hobbs M. & Meyn R. E. Clonogenic cell survival assay. Methods Mol Med 110, 21–28 (2005). 15901923 10.1385/1-59259-869-2:021

[b18] Castedo M. . Quantitation of mitochondrial alterations associated with apoptosis. J Immunol Methods 265, 39–47 (2002). 12072177 10.1016/s0022-1759(02)00069-8

[b19] Murakami Y. . Inhibition of nuclear translocation of apoptosis-inducing factor is an essential mechanism of the neuroprotective activity of pigment epithelium-derived factor in a rat model of retinal degeneration. Am J Pathol 173, 1326–1338 (2008). 18845835 10.2353/ajpath.2008.080466 PMC2570123

[b20] Jemaa M. . Selective killing of p53-deficient cancer cells by SP600125. EMBO Mol Med 4, 500–514 (2012). 22438244 10.1002/emmm.201200228 PMC3443949

[b21] Tan A. S. & Berridge M. V. Superoxide produced by activated neutrophils efficiently reduces the tetrazolium salt, WST-1 to produce a soluble formazan: a simple colorimetric assay for measuring respiratory burst activation and for screening anti-inflammatory agents. J Immunol Methods 238, 59–68 (2000). 10758236 10.1016/s0022-1759(00)00156-3

[b22] Jin Z. & El-Deiry W. S. Overview of cell death signaling pathways. Cancer Biol Ther 4, 139–163 (2005). 15725726 10.4161/cbt.4.2.1508

[b23] Brunelle J. K. & Letai A. Control of mitochondrial apoptosis by the Bcl-2 family. J Cell Sci 122, 437–441 (2009). 19193868 10.1242/jcs.031682 PMC2714431

[b24] Elmore S. Apoptosis: a review of programmed cell death. Toxicol Pathol 35, 495–516 (2007). 17562483 10.1080/01926230701320337 PMC2117903

[b25] Lang F. . Eryptosis, a window to systemic disease. Cell Physiol Biochem 22, 373–380 (2008). 19088418 10.1159/000185448

[b26] Abed M. . Sphingomyelinase-induced adhesion of eryptotic erythrocytes to endothelial cells. Am J Physiol Cell Physiol 303, C991–999 (2010). 10.1152/ajpcell.00239.2012 22954799

[b27] Berg C. P. . Human mature red blood cells express caspase-3 and caspase-8, but are devoid of mitochondrial regulators of apoptosis. Cell Death Differ 8, 1197–1206 (2001). 11753567 10.1038/sj.cdd.4400905

[b28] Bratosin D. . Active caspases-8 and -3 in circulating human erythrocytes purified on immobilized annexin-V: a cytometric demonstration. Cytometry A 75, 236–244 (2009). 19061248 10.1002/cyto.a.20693

[b29] Antonelou M. H. . Red blood cell aging markers during storage in citrate-phosphate-dextrose-saline-adenine-glucose-mannitol. Transfusion 50, 376–389 (2010). 19874562 10.1111/j.1537-2995.2009.02449.x

[b30] Mandal D., Moitra P. K., Saha S. & Basu J. Caspase 3 regulates phosphatidylserine externalization and phagocytosis of oxidatively stressed erythrocytes. FEBS Lett 513, 184–188 (2002). 11904147 10.1016/s0014-5793(02)02294-9

[b31] Mandal D., Mazumder A., Das P., Kundu M. & Basu J. Fas-, caspase 8-, and caspase 3-dependent signaling regulates the activity of the aminophospholipid translocase and phosphatidylserine externalization in human erythrocytes. J Biol Chem 280, 39460–39467 (2005). 16179347 10.1074/jbc.M506928200

[b32] Antonelou M. H., Kriebardis A. G. & Papassideri I. S. Aging and death signalling in mature red cells: from basic science to transfusion practice. Blood Transfus 8 Suppl 3, s39–47 (2010). 20606748 10.2450/2010.007S PMC2897187

[b33] Kriebardis A. G. . Storage-dependent remodeling of the red blood cell membrane is associated with increased immunoglobulin G binding, lipid raft rearrangement, and caspase activation. Transfusion 47, 1212–1220 (2007). 17581156 10.1111/j.1537-2995.2007.01254.x

[b34] Walker B. . Dynamic adhesion of eryptotic erythrocytes to immobilized platelets via platelet phosphatidylserine receptors. Am J Physiol Cell Physiol 306, C291–297 (2014). 24284794 10.1152/ajpcell.00318.2013

[b35] Pretorius E. . Eryptosis as a marker of Parkinson’s disease. Aging (Albany NY) 6, 788–819 (2014). 25411230 10.18632/aging.100695 PMC4247384

[b36] Lang F., Abed M., Lang E. & Foller M. Oxidative stress and suicidal erythrocyte death. Antioxid Redox Signal 21, 138–153 (2014). 24359125 10.1089/ars.2013.5747

[b37] Senderowicz A. M. Cell cycle modulators for the treatment of lung malignancies. Clin Lung Cancer 5, 158–168 (2003). 14667271 10.3816/CLC.2003.n.028

[b38] Hwang H. C. & Clurman B. E. Cyclin E in normal and neoplastic cell cycles. Oncogene 24, 2776–2786 (2005). 15838514 10.1038/sj.onc.1208613

[b39] Castedo M., Perfettini J. L., Roumier T. & Kroemer G. Cyclin-dependent kinase-1: linking apoptosis to cell cycle and mitotic catastrophe. Cell Death Differ 9, 1287–1293 (2002). 12478465 10.1038/sj.cdd.4401130

[b40] Jacobberger J. W. . A new biomarker for mitotic cells. Cytometry A 73, 5–15 (2008). 18061938 10.1002/cyto.a.20501

[b41] Russell F. E. Weever fish sting: the last word. Br Med J (Clin Res Ed) 287, 981–982 (1983). 10.1136/bmj.287.6397.981-c PMC1549177 6412912

