# Supplementary material for: Purified Lesser weever fish venom (Trachinus vipera) induces eryptosis, apoptosis and cell cycle arrest
Source: Sci Rep. 2016 Dec 20;6:39288. doi: 10.1038/srep39288 (PMC5171788; doi:10.1038/srep39288)
Supplement: Supplementary Figure S1 [file srep39288-s1.pdf]

Purified Lesser weever fish venom (*Trachinus vipera*) induces eryptosis, apoptosis and cell cycle arrest.

Myriam Fezai[1-3], Chaker Slaymi[4,5], Mossadok Ben-Attia[1,2], Florian Lang[3*] and Mohamed Jemaà[3*]

[1]Laboratory of Biomonitoring of the Environment (LR01/ES14), Faculty of Sciences of Bizerte, Tunis street, 7021 Zarzouna, Bizerte, Tunisia.

[2]University of Carthage, Amilcar avenue 77, 1054 Tunisia.

[3]Department of Cardiology, Vascular Medicine and Physiology, University of Tuebingen, Gmelinstr. 5/Otfried-Mueller-Str. 10, D-72076 Tuebingen, Germany.

[4]Centre de Recherche de Biochimie Macromoléculaire - CNRS, UMR 5237, Mende 1919, 34293 Montpellier, France.

[5]University of Montpellier, Auguste Broussonet street 163, 34090 Montpellier, France.

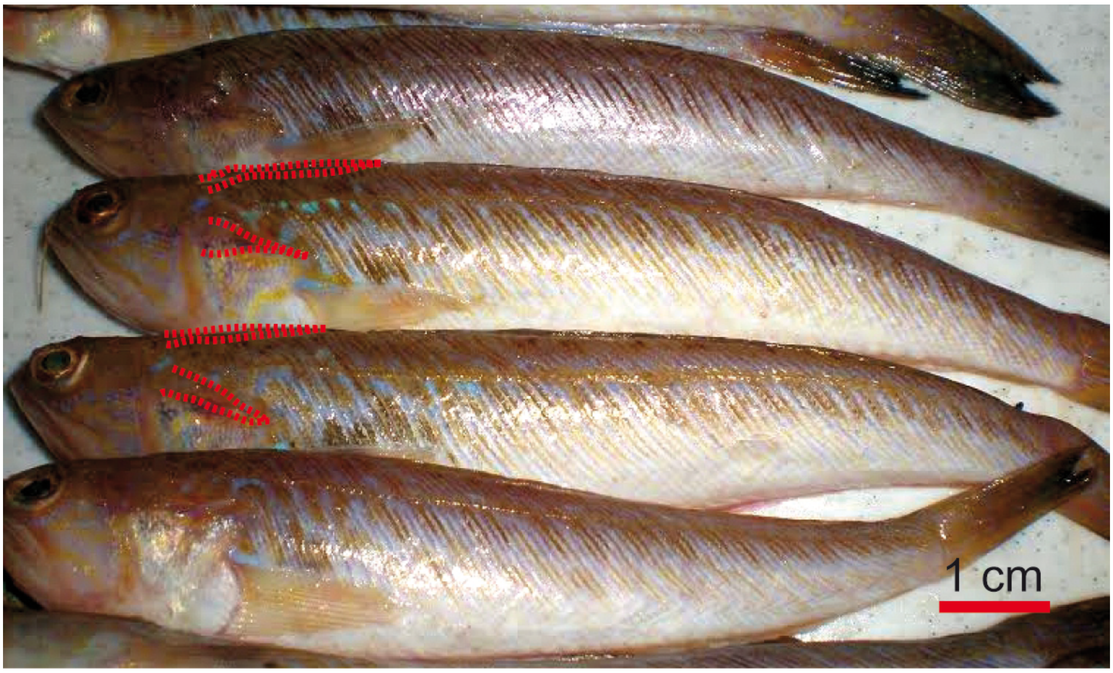

Figure S1. The *Trachinus vipera*.

The captured lesser fish on the Tunisian coast with schematic localization of the poisonous spines (interrupted red lines) at the anterior back fin and the base of the spine located on the gill cover.
